# Roles of Factor XII in Innate Immunity

**DOI:** 10.3389/fimmu.2019.02011

**Published:** 2019-08-22

**Authors:** Thomas Renné, Evi X. Stavrou

**Affiliations:** ^1^Institute of Clinical Chemistry and Laboratory Medicine, University Medical Center Hamburg-Eppendorf, Hamburg, Germany; ^2^Section of Hematology-Oncology, Department of Medicine, Louis Stokes Cleveland Veterans Administration Medical Center, VA Northeast Ohio Healthcare System, Cleveland, OH, United States; ^3^Hematology and Oncology Division, Department of Medicine, Case Western Reserve University School of Medicine, Cleveland, OH, United States

**Keywords:** factor XII, uPAR, contact activation, innate immunity, sepsis, neutrophil extracellular traps, cancer progression, wound healing

## Abstract

Factor XII (FXII) is the zymogen of serine protease, factor XIIa (FXIIa). FXIIa enzymatic activities have been extensively studied and FXIIa inhibition is emerging as a promising target to treat or prevent thrombosis without creating a hemostatic defect. FXII and plasma prekallikrein reciprocally activate each other and result in liberation of bradykinin. Due to its unique structure among coagulation factors, FXII exerts mitogenic activity in endothelial and smooth muscle cells, indicating that zymogen FXII has activities independent of its protease function. A growing body of evidence has revealed that both FXII and FXIIa upregulate neutrophil functions, contribute to macrophage polarization and induce T-cell differentiation. *In vivo*, these signaling activities contribute to host defense against pathogens, mediate the development of neuroinflammation, influence wound repair and may facilitate cancer maintenance and progression. Here, we review the roles of FXII in innate immunity as they relate to non-sterile and sterile immune responses.

## Introduction

Factor XII (FXII) is the zymogen of serine protease, factor XIIa (FXIIa). FXII is converted to its active enzyme (FXIIa) by plasma kallikrein (PKa) or by its unique ability to auto-activate following binding to artificial or biologic surfaces (1). *In vivo*, FXIIa initiates coagulation via the fibrin-forming “intrinsic” pathway and promotes inflammation via the bradykinin (BK)-producing kallikrein kinin system comprising, high molecular weight kininogen (HK) and plasma prekallikrein (PK). Together, FXII, PK, and HK are termed plasma contact activation system. Furthermore, FXIIa may modulate components of the complement and fibrinolytic systems however to date, these activities have only been demonstrated *in vitro*. There has been renewed interest in FXII due to the recognition that several substances [e.g., polyphosphate, misfolded protein, vascular collagen, DNA in neutrophil extracellular traps (NETs)] support FXII auto-activation *in vivo* (2–7) and prior studies showing that FXII deficient (*F12*^−/−^) animals are protected from thrombosis without impaired hemostasis (8–11). Similarly, pharmacologic targeting of FXII or FXIIa provided protection from thrombosis without increased incidence of bleeding (7, 12).

Although the enzymatic activities of FXII have been thoroughly studied and the FXIIa protease domain structure is known (13), few zymogen FXII functions have been recognized. Prior reports have shown that FXII deficiency is linked to decreased infiltration of inflammatory cells into skin windows (14). In human plasma, FXII and FXIIa promote neutrophil aggregation and degranulation (15) and FXII with related proteins assembles on the surface of neutrophils (16). FXII has two epidermal growth factor domains that contribute mitogenic activity in endothelial and smooth muscle cells (17, 18), similar to activated Factor X (FXa), protein C and S (19–21). These data indicate that zymogen FXII differentially regulates cell activities independent of its protease function.

Despite its role in pathologic thrombosis, FXII deficiency is rare in humans indicating that it may contribute to homeostatic functions. Here, we review the role of FXII in non-sterile and sterile inflammation. We focus on its differential contribution to host immune responses to infectious pathogens and contrast these to newly appreciated FXII functions in chronic, sterile inflammation.

## Role of Factor XII in Infectious Settings

### FXIIa Interactions With Bacteria

Experimental and clinical studies have shown a link between FXII and the contact system to infections (22–25). Several mechanisms have been proposed by which contact system components modulate infectious burden and the inflammatory response. *First*, FXII directly binds to the surface of bacteria, fungi, viruses as well as on neutrophils and neutrophil extracellular traps (NETs) where it autoactivates (26). In a *Salmonella*-induced pneumonia model in rats, targeting FXIIa using the peptide inhibitor H-D-Pro-Phe-Arg-chloromethylketone ameliorated lung injury and largely prevented bacteria-induced bleeding (27). Another mechanism by which microorganisms can induce FXII zymogen activation, involves contact activation by the inorganic polymer polyphosphate (polyP). Bacterial-derived polyphosphate, similar to polymers exposed by activated platelets, drives contact system activation (2). Microbes contain polyP that serves as their energy storage pool, with a size varying in length from hundreds to thousands of phosphate units (28). PolyP forms calcium ion-rich nanoparticles that trigger FXII autoactivation independently of polymer length on cell surfaces *in vivo* (29, 30). Consistent with this finding, polyP from *Salmonella* and *E. coli* species shows extreme potency at triggering the contact pathway (2, 30). PolyP not only contributes to FXIIa-mediated fibrin formation, but it also interferes with binding of fibrin to tissue-type plasminogen activator or plasminogen (31), thus affecting fibrinolysis. Waack et al. (32) showed that metalloprotease CpaA secreted from *Acinetobacter baumanii* species inactivates FXII. The cleavage by CpaA was mapped on two positions, 279–280 and 308–309, within the proline-rich region of FXII and cleavage at the 308–309 site was a requirement for inactivation of FXII. At both sites, cleavage takes place between a proline and an O-linked glycosylated threonine residue and deglycosylation of FXII rescues from CpaA cleavage. Strikingly, mutant FXII (Thr309Lys) from patients with hereditary angioedema type III (HAEIII), where O-linked glycosylation at position 309 is lost and inactivation by C1 esterase inhibitor during activation by plasmin is reduced (33, 34), is protected from CpaA inactivation (32). By inactivating FXII, CpaA attenuates important coagulation and inflammatory mechanisms, thus allowing *A. baumannii* dissemination (32). To date, the structural details of the interaction(s) between FXII and pathogens remain elusive. An exciting possibility would be that FXII functions as a soluble pattern recognition molecule or damage-associated molecular pattern (DAMP), binding to defined sites on microorganisms. Until such time that the surface-binding domains of FXII are fully characterized, this scenario remains speculative.

### FXIIa-Initiated Bradykinin Formation

As discussed above, bacteria can activate the contact system either through direct binding or indirectly through the release of mediators (35). *Staphylococcus aureus* bacteria release proteinases which directly cleave HK whereas other proteinases such as from *Porphyromonas gingivalis*, indirectly lead to HK cleavage through proteolytic activation of FXII (36). HK cleavage results in liberation of BK, a potent proinflammatory and vasodilatative peptide (37). BK regulates vascular smooth muscle relaxation, increases vascular permeability and can directly influence leukocyte functions (38, 39). Host infection with *hantavirus* results in increased enzymatic activities of FXIIa and PKa and subsequent release of BK, leading to enhanced endothelial cell permeability and vascular leak (40). Another virus HSV1 directly binds to FXII and in the presence of PKa, promotes its activation. Inhibiting FXIIa or use of antibodies to FXII, PK, and Factor XI prevented HSV1-initiated clotting (41). Persistent bacteria-mediated contact system activation may result in abnormally high plasma BK levels and consumptive coagulopathy and can induce hypotension and edema, contributing to multi-organ failure. Indeed, in a model of lethal *E. coli* bacteremia in baboons, FXII activation was related with the hypotension seen in these animals (42). In this primate model, contact activation and FXIIa generation manifested primarily by a significant decrease in HK and a significant increase in PKa, indicative of increased BK formation. Animals treated with C6B7, a monoclonal antibody to FXII, experienced an initial drop in systemic blood pressure that subsequently resolved and extended their overall survival (42). However, all groups including C6B7-treated animals, equally developed disseminated intravascular coagulation (DIC) manifesting with thrombocytopenia, hypofibrinogenemia and decreased factor V levels (42). Interfering with BK activity resulted in attenuation of acute respiratory distress syndrome (ARDS) features in a rat hypothalamic nuclei lesion model (43) and significantly improved outcomes of patients with systemic inflammatory response syndrome caused by Gram-negative bacteria (44). These data indicate that interfering with FXIIa-induced BK formation, alone can have beneficial effects in host defense.

The protective effects of functional FXII deficiency were shown to be pathogen-specific (45). Stroo et al. showed that genetic ablation of FXII conferred a survival advantage from *Klebsiella pneumoniae* sepsis but not from *Streptococcus pneumoniae* sepsis (45). The authors postulate that the contact system becomes operative in late disease stages, where damage due to overwhelming inflammation accounts for FXII activation and BK release (45). It is important to note however that in this study, *Klebsiella*-infected FXII deficient mice also had consistently decreased bacterial loads. This finding raises the intriguing possibility that FXII may preferentially interact with specific pathogens or their soluble mediators to facilitate bacterial maintenance and invasion. Further studies are warranted to mechanistically determine if under certain infectious settings, FXII serves as an *in vivo* bacterial “fitness factor.”

### FXII Responses Independent of Plasma Kallikrein

Although BK is the main inflammatory mediator of the contact system, FXIIa also initiates the classical complement cascade (46) and exerts direct proinflammatory properties *in vitro*. In the presence of lipopolysaccharide (LPS), FXII exposure of peripheral blood monocytes resulted in enhanced interleukin 1 (IL-1) activity and LPS was also essential for FXII- and FXIIa-triggered expression of IL-6 and IL-23 by splenic dendritic cells (47). Moreover, zymogen FXII and FXIIa were shown to promote neutrophil degranulation (15) and FXII content in bronchoalveolar lavage fluid from ARDS patients, was significantly higher in non-survivors than in survivors (48). In this setting, FXII but not FXIIa, upregulated the expression of inflammatory cytokines interleukin 8 (IL-8), IL-1β, interleukin 6 (IL-6), CXC chemokine ligand 5 (CXCL5), leukemia inhibitory factor (LIF), and tumor necrosis factor alpha (TNF-α), independently of plasma kallikrein (48).

**Figure 1:**
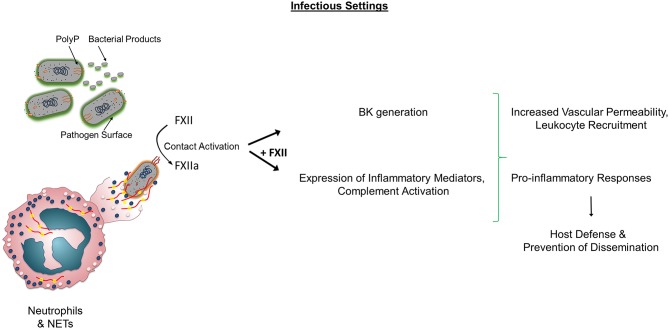
Role of FXII in non-sterile inflammation. In infectious conditions, Factor XII (FXII) activation occurs through various mechanisms including pathogen surfaces, bacterial products, polyP, neutrophils, and NETs. Activated FXII (FXIIa) leads to BK generation and activates components of the complement system. Both FXII and FXIIa increase the expression of pro-inflammatory cytokines and influence leukocyte functions. In certain settings, the sum of these activities contributes to prevention of dissemination. Alternatively, the presence of FXII and subsequent FXIIa and BK generation, lead to adverse outcomes and increased mortality independently of Factor XI activation. PolyP, polyphosphate; BK, Bradykinin; NETs, Neutrophil Extracellular Traps.

In sum, the aforementioned studies highlight that FXII has a complex role in sepsis eliciting distinct pro-coagulant and pro-inflammatory responses that differentially contribute to infectious outcomes (Figure 1).

## Role of FXII in Sterile Inflammation

Discoveries over the past few years have increased our understanding of the diverse FXII functions. Here, we summarize the signaling events for FXII and FXIIa in innate immune cells with a focus on sterile inflammatory conditions and discuss the downstream physiologic and pathologic consequences.

### Immune Cell Biology of FXII and FXIIa

Our lab recently reported on the role of FXII in neutrophil proinflammatory responses (49). We showed that autocrine FXII binds to urokinase plasminogen activator receptor (uPAR) with high affinity and their interaction induces Akt2 phosphorylation. *In vivo*, FXII (which interacts with domain II of uPAR) and HK (which binds to domains II and III), compete for binding and their interaction with uPAR is mutually exclusive (50–52). Moreover, in resting neutrophils uPAR is stored intracellularly secretory vesicles and specific granules (53). Neutrophil activation results in upregulation of uPAR expression and its subsequent translocation to the neutrophil plasma membrane (53). This highly regulated appearance of FXII binding sites on primed neutrophils, partly explains why the FXII-uPAR interaction is not operating continuously. Another rate-limiting step for FXII and uPAR binding is FXII's cell-binding ability. *In vivo*, FXII binding to cells is augmented when the local *free* zinc ion concentration rises significantly from baseline plasma levels of ~20 nM (54, 55) to a micromolar (μM) range. The source of extracellular zinc was previously shown to derive from activated cells such as platelets (50, 56). Neutrophils also contain a rich network of zinc transporters and may potentiate the mobilization of zinc toward the extracellular compartment during inflammation. To this end, surface plasmon resonance confirmed that in the absence of zinc, FXII does not interact with uPAR (49). In sum, FXII-uPAR complex formation is a highly regulated process *in vivo* governed both by uPAR surface expression and the local concentration of zinc ions (49).

**Figure 2:**
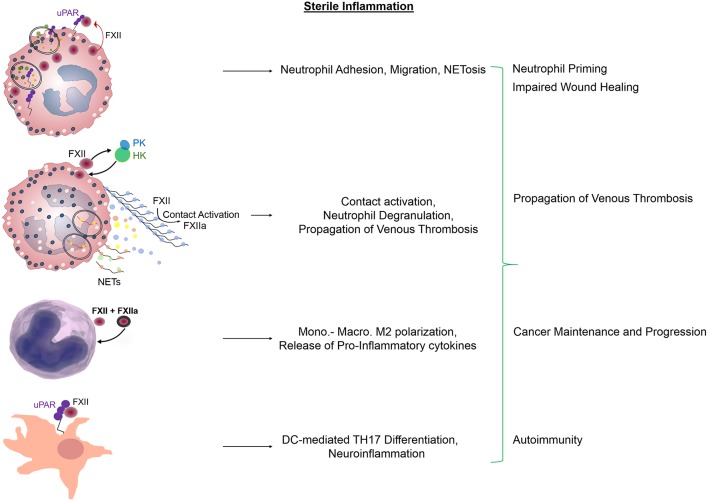
Regulatory functions of FXII and FXIIa in sterile inflammation. Zymogen FXII functions as an autocrine messenger through uPAR to promote Akt2S^474^ phosphorylation. Propagation of FXII-mediated neutrophil activities includes adhesion, chemotaxis that leads to neutrophil trafficking at sites of inflammation and NET formation. Subsequent contact activation on the surface of preformed NETs leads to FXIIa generation and fibrin formation. In monocytes and macrophages, FXII and FXIIa upregulate the expression of pro-inflammatory mediators and promote cell polarization toward an M2 phenotype. In dendritic cells, FXII signals through uPAR to induce the differentiation of naive T-cells to TH17 cells. These immune cell responses contribute to impaired wound healing, propagation of venous thrombosis, tumor maintenance, and invasion as well as tissue damage during CNS autoimmunity. NETs, neutrophil extracellular traps; Mono, Monocytes; Macro, Macrophages; DC, Dendritic cells; TH17, T-helper cells.

Having no intracellular domain, uPAR has to engage other membrane receptors that mediate signals to cells. uPAR has an intimate relationship with integrins regulating their affinity and avidity, but the reverse is also true, integrins are able to modulate the activity of uPAR. In neutrophils and macrophages, this crosstalk permits an indirect connection of uPAR with the integrin interactome (57). In this context, we found that the FXII-uPAR interaction increases the surface expression of neutrophil αMβ2 integrin, leads to intracellular Ca^2+^ mobilization, and promotes histone citrullination (49). These signaling events, which are independent of FXII enzymatic activity, upregulate neutrophil functions. Specifically, FXII-uPAR promote neutrophil adhesion to extracellular matrices including fibrinogen, increase directional cell migration (chemotaxis) and NET formation (49) (Figure 2). The identification that FXII itself promotes NET formation is novel and therapeutically relevant since the conventional thinking was that their relationship lies purely on activation of circulating FXII on preformed NETs (5). On-going studies in our lab seek to determine if FXII-uPAR-mediated functions in neutrophils are exclusively initiated through αMβ2 or if additional lateral partners are involved in intracellular signal transduction.

Eventually, neutrophil priming enables circulating FXII to bind onto neutrophils and autoactivate (5, 49). In addition to FXII, HK and PK also assemble on the surface of primed neutrophils (16) via binding to proteoglycans (58), which modulate the liberation of BK (59). This neutrophil-bound contact system generates FXIIa which selectively induces neutrophil aggregation and degranulation (15). FXIIa-mediated activation of PK and/or release of neutrophil-derived tissue kallikrein, may be responsible for the circumscribed formation of BK. The local release of BK in turn may facilitate the escape of neutrophils toward the extracellular space by causing endothelial cell retraction, and permits transudation of plasma content by controlling vascular permeability at sites of inflammation (Figure 2).

In addition to neutrophil pro-inflammatory responses, FXII modulates the functions of an array of innate immune cells (47, 60, 61). Previous studies have shown that macrophages react to FXII and FXIIa, by increasing the production of IL-6, IL-12, and tumor necrosis factor (TNF)-α. Moreover, peripheral blood mononuclear cells treated with FXII acquire an M2-tissue reparative phenotype as seen by increased secretion of IL-4, IL-8, IL-10, and transforming growth factor (TGF)-β. Finally, FXII was shown to promote the differentiation of T helper (Th) naive cells to TH17 cells (62) (Figure 2).

Collectively, these data highlight two key concepts: (i) zymogen FXII is able to directly influence innate immune functions, and (ii) FXII and FXIIa exert distinct and separate cellular effects.

## Influence of FXII and FXIIa on Sterile Inflammatory Tissue Responses

Here, we focus on how FXII- and FXIIa-mediated responses in innate and adaptive immune cells are integrated in the activation, regulation and effector mechanisms that lead to diverse pathologies and extend beyond their role in contact activation.

### FXII-Neutrophil Crosstalk in Wound Healing and Tissue Disrepair

Wound healing is an intricate process that consists of multiple phases, each of which is indispensable for proper tissue repair. Prompt initiation and resolution of each wound healing phase namely, hemostasis, inflammation, proliferation, and tissue remodeling, is critical for promoting repair and avoiding scar formation. While neutrophils play a central role in the inflammatory phase, recent evidence suggests that their prompt removal is equally important for orderly wound progression through subsequent phases of healing (63). Animal models show that excess neutrophil entry into wound sites interferes with keratinocyte proliferation and migration (64). In addition to these effects, lengthy neutrophil presence in wounds leads to unrestricted proteolytic activity by neutrophil granular enzymes that eventually leads to a pathologic chain of events leading to matrix degradation and proteolytic inactivation of growth factors and their receptors (65). Indeed, proteomic studies showed that levels of neutrophil granular enzymes, among them neutrophil elastase, are increased in the exudate of non-healing human wounds and is thought to reflect a chronic, inflammatory, tissue-destructive microenvironment (66). In contrast, levels of alpha1-antitrypsin, the physiologic inhibitor of neutrophil elastase, were increased in well-healing wounds (65). Neutrophil elastase is a critical component of NETs and contributes to their function (67). Recent studies show that circulating neutrophils from diabetic individuals are primed constitutively to produce NETs (68), and NETosis delayed diabetic wound healing in mice and humans (69). Therefore, continued recruitment, or buildup of active neutrophils, inevitably prolongs inflammation and contributes to the development of chronic wounds.

In two models of sterile inflammation, we show that neutrophil recruitment is decreased in FXII deficient (*F12*^−/−^*)* mice and this is a bone marrow-endowed function (49). Decreased neutrophil migration at sites of inflammation was associated with reduced NETs into the wound microenvironment and improved wound healing (49). Altogether, these data support that limiting the activity of neutrophils may be beneficial for the treatment of recalcitrant wounds and provide the rationale for our on-going studies, harnessing the FXII-uPAR axis in neutrophils as a therapeutic strategy to promote wound healing. Given that loss of uPA but not uPAR delays wound healing (70), our findings confirm prior studies that the influence on wound healing is ligand dependent, not uPAR dependent.

### The FXII-uPAR Axis in Cancer Maintenance and Progression

We previously published our findings on the contribution of FXII and polyP in prostate cancer-associated thrombosis (6). As FXII and FXIIa each exhibit growth factor properties, the question arises as to their potential involvement in regulating cancer cell behavior. uPAR is emerging as a cell surface-associated receptor that contributes to the development, progression, maintenance and metastasis of several cancers including epithelial ovarian cancer (EOC) (71–73). In EOC cells, uPAR has been reported to be overexpressed in more than 90% of ovarian cancer patients whereas, it is absent or minimally expressed in normal ovarian surface epithelium (74). uPAR overexpression in human ovarian cancer is associated with decreased overall survival (71–73). Moreover, global gene expression analysis revealed increased levels of *F12* mRNA in EOC tumors but not in normal ovarian epithelium or fallopian tubes (75), and stimulation of ovarian cancer cells with FXII induced cell invasion (76). Interestingly, neutrophils have also recently been recognized to be important players in the ovarian tumor microenvironment, inducing epithelial-to-mesenchymal transition and promoting tumor invasion (53, 77, 78) (Figure 2). In this framework, ascertaining the role of host vs. tumor FXII-uPAR in EOC progression is likely to be a promising line of investigation.

### FXII and Thromboinflammation

In a traditional view, the pathogenesis of DVT can be captured by Virchow's triad, which postulates that three main factors contribute to VTE development: reduced blood flow (stasis), vascular endothelial damage, and a hypercoagulable state. This conventional paradigm dramatically shifted with the observation in recent years that neutrophils significantly contribute to thrombosis, termed thromboinflammation (5, 79, 80). The cooperation of platelets with neutrophils was identified using a murine model of DVT in which flow restriction induces thrombosis in the inferior vena cava (IVC) (5). In this model, platelets and neutrophils are promptly recruited to the vessel wall within hours of reduced blood flow and engage in heterotypic cell-cell interactions (5). These interactions facilitate DVT growth and propagation by: (1) supporting additional neutrophil recruitment; and (2) stimulating neutrophils to release NETs which are indispensable for subsequent DVT propagation through FXII autoactivation, coagulation factor assembly and fibrin formation (5) (Figure 2). Exposure of endogenous polyP on activated platelets or intravenous administration of polyP resulted in FXII activation and lethal pulmonary embolism in wild type mice. In contrast, genetic FXII deficiency or a FXIIa inhibitor (2, 81), prevented the development of PE. Recombinant Ixodes ricinus contact phase inhibitor (Ir-CPI), a Kunitz-type protein expressed by the tick Ixodes ricinus, specifically interacts with human FXIIa, FXIa, and PKa and results in prolongation of the aPTT *in vitro* (81). Intravenous administration of IrCPI in rats and mice caused rescued animals from venous thrombus formation in a dose-dependent fashion (81).

While multiple models have demonstrated the critical role of both FXII and neutrophils in thrombosis, some exceptions seem to exist. FXII and neutrophils were recently shown to be dispensable for vascular occlusion in the large veins of the head following combined “knockdown” of natural anticoagulants, protein C and antithrombin (82). However, unlike the IVC stenosis model which mimics human DVT by reducing venous return, the study by Heestermans et al. involved large alterations in the natural plasma protease inhibitor balance. This setting appears to be more reflective of a state of disseminated intravascular coagulation (DIC) with consumptive drop in platelet count, spontaneous intravascular thrombosis and tissue fibrin deposition, rather than DVT forming as a result of venous stasis in the absence of a severe hypercoagulable state. Although additional studies are required to demonstrate the clinical utility of FXIIa inhibitors in the management of DVT, it will be interesting to also examine whether there is potential for FXII inhibitors that interfere with zymogen FXII activation of cells, as a strategy to mitigate DVT development.

### Influence of FXII in Autoimmunity

It is widely accepted that multiple sclerosis develops due to peripheral autoreactive T-cells which are able to pass through the blood–brain barrier, induce diffuse inflammatory lesions in the brain, thus leading to demyelination. Prior studies have shown that effector T-helper cells (TH1 and TH17) play a key role in the development of inflammation and tissue damage during CNS autoimmunity (83–85). Interaction of T cells with dendritic cells (DCs), antigen-presenting cells (APCs), is crucial for proper T-cell differentiation (86, 87). Excess presence of effector T-cells in brain lesions and increased levels of T-cell-derived cytokines in peripheral blood mononuclear cells (PBMCs) of patients also highlight the causal role of autoreactive T-cells in human MS (88, 89). Gobel et al. investigated the role of FXII in autoimmunity using an experimental model of MS (62). They show that *F12*^−/−^ mice are less prone to developing CNS inflammation and FXII worsened features of experimental autoimmune encephalomyelitis (EAE). Pharmacologic blockade of FXIIa was protective against EAE, independently of Factor XI and kallikrein-kinin generation (62). Mechanistically, FXII stimulated DC-induced TH17-cell generation in a uPAR-dependent manner. These results increased our understanding on the role of FXII in adaptive immunity and its potential as a therapeutic target in autoimmune disease states (Figure 2).

## Conclusion

In summary, new perspectives have emerged that call for a reappraisal of the role of FXII *in vivo*. Far beyond its fundamental property to autoactivate, FXII engages in complex receptor interactions that cooperatively influence immune cell behavior and contribute to physiologic and pathophysiologic responses. The details of the signaling pathways downstream of FXII are still incomplete. The challenge now is to elucidate the subtle molecular details of this versatile orchestrator and its impact on *in vivo* processes that determine health and disease.

## Author Contributions

TR and ES wrote and edited all versions of the manuscript.

### Conflict of Interest Statement

The authors declare that the research was conducted in the absence of any commercial or financial relationships that could be construed as a potential conflict of interest.
